# Person-Environment Fit and Socioeconomic Status in Medical School

**DOI:** 10.1007/s40670-024-02174-x

**Published:** 2024-10-03

**Authors:** Regina G. Russell, Mytien Nguyen, Catherine Havemann, Alexis Webber, Jon Andre Parilla, Alejandra Casillas, Dowin Boatright, Hyacinth Mason

**Affiliations:** 1 Vanderbilt University School of Nursing, Nashville, TN USA; 2 MD/PhD Program, Yale School of Medicine, New Haven, CT 06510 USA; 3 Section of Emergency Medicine, University of Chicago, Chicago, IL USA; 4 Department of General Surgery, Albany Medical Center, Albany, NY USA; 5 Kirk Krekorian School of Medicine at University of Nevada, Las Vegas, NV USA; 6 Division of General Internal Medicine and Health Services Research, Department of Medicine, UCLA David Geffen School of Medicine, Los Angeles, CA USA; 7 Ronald O. Perelman Department of Emergency Medicine, Emergency Medicine and Population Health, NYU Grossman School of Medicine, New York, NY USA; 8 Tufts University School of Medicine, Boston, MA USA

**Keywords:** Diversity, Medical students, Qualitative research, First-generation college students, Low-income learners, Learning environments

## Abstract

**Purpose:**

This study examined the impact of socioeconomic status (SES) on medical education in the context of person-environment fit (PE fit) theory, and specifically focused on the medical school experiences of students from lower-SES backgrounds.

**Method:**

A constructivist approach was used in this qualitative study of 48 medical students from 27 US medical schools, all of whom self-identified as first-generation college graduates and/or being from a lower-income background (30 were both). Semi-structured audio-only interviews were conducted with these demographically and geographically diverse students from November 2021 through April 2022. Themes were identified using open coding and content analysis software.

**Results:**

Almost all, 44 of 48, interviews included themes related to PE fit. Medical students indicated three interacting domains in which PE fit is relevant for them: (1) school, (2) clinical, and (3) professional environments. Learners from lower-SES backgrounds describe struggling to navigate multiple environments that are unfamiliar, culturally complex, and both personally and financially costly. They also describe ways they are addressing gaps, generating positive changes, supporting underserved patients, and broadening the perspectives of peers and educators.

**Conclusions:**

PE fit theory provides a lens to understand unique aspects of lower-SES medical student experiences, including navigation of professional identity formation. It is critical for medical schools, funders, peers, and professional communities to sustain learning environments that support the flourishing of medical students from lower-SES backgrounds. This support includes transferring the burden of addressing fit from individual learners and marginalized classes of learners to educational, clinical, and professional organizations.

**Supplementary Information:**

The online version contains supplementary material available at 10.1007/s40670-024-02174-x.

## Background

### Person-Environment Fit Theory

When a person feels that they do not belong in a certain environment, such as medical school, it can affect performance, retention, and satisfaction [1]. Person-environment fit (PE fit) theory addresses the consequences of a person’s environment being aligned or misaligned; and has been commonly applied to employment and organizational settings [2–5]. In the healthcare realm, PE fit theory was first applied to explore the relationships between geriatric patients’ changing functional abilities, the characteristics of their environments, and their quality-of-life outcomes [6–11]. The goal of PE fit theory in industrial–organizational psychology is to match people to employment and develop their careers [12–16]. A common goal in the healthcare occupational realm has been to explore factors affecting medical professionals’ job satisfaction, including identifying individual and organizational characteristics linked to PE fit [17–19]. Past studies have suggested that this theory can also be used to understand the varied experiences of higher education students [20–22].

Our aim in this study was to apply PE fit theory to the medical education sphere, and specifically to explore common themes applicable to the medical school experiences of learners from lower-socioeconomic status (SES) backgrounds. For this study, PE fit theory is defined as the interactions between an individual’s characteristics and their environments, whereby environments can affect that individual’s motivation, performance, self-esteem, mental health, and other personal characteristics. Of course, individuals also affect their environments [23]. PE fit theorists try to describe and build conceptual models for core features of the complex relationships between individuals and their environments.

### Socioeconomic Status (SES)

Group status-sorting factors related to income, education, and career bestow a level of social or cultural privilege on individuals in any given environment. Most medical students in the USA were raised in upper- or middle-income families and have parents who possess at least 4-year college degrees [24, 25]—thus having greater access to educational opportunities than do people from lower-SES families. Indeed, parental experience with higher education is protective against medical school attrition [26].

Patient populations are currently much more racially, ethnically, and economically diverse than their doctors. The medical education community has emphasized its commitment to diversifying the physician workforce to meet diverse patient needs, address social accountability, and pursue health equity [27]. However, attracting diverse learners can lead to a mismatch between traditional learning environments and the expectations of these newly expanded student populations. An individual learner may experience this as a misfit between their own goals and what the learning environment, or profession, has to offer them. PE fit theory can be used to illuminate the disparate experiences of underrepresented students in medical education [20–22].

### Medical Education and Professional Identity Formation

Medical education is a long and impactful process that involves moving from the periphery to the center of caring for others [28]. Learners are expected to develop a professional identity along with the knowledge and skills necessary to be an excellent physician. A recent study by Kay et al. demonstrated that medical students experience cognitive disequilibrium and the need to adapt their mental models at key points in medical school [29]. While these experiences are necessary catalysts for professional identify formation, they can also be very difficult and leave students needing additional support [29–32].

Cognitive changes due to environment pressures are part of the normal developmental process. However, PE fit research demonstrates that one’s “environmental press” can be more or less intense [33]. Students with backgrounds or characteristics that are very divergent from the norm are likely to experience a more extreme and continuous environmental press—a stress-inducing mismatch between themselves and their environments.

The perceived match for students with different environments and specialty cultures can be helpful to explore in career advising. The Association of American Medical College’s Careers in Medicine program provides guidance to students in order to identify the best specialty for that person’s interests and abilities [34]. They found in a 2022 study that the most important factors to medical students considering resident placements were “fit” with personality, interests, and skills, along with content knowledge of the specialty [35].

Concerns have been raised regarding the use of the term “fit” in selection processes due to histories of exclusion and bias in medical school admissions and residency selections [36]. However, it is important to note that “fit” has a broader meaning and includes both the preferences of learners and the characteristics of programs [37]. The concept of fit can also be used to illuminate specific ways training programs can evolve to become more welcoming, inclusive, and fair to diverse learners. The concept of fit represents a deep vein of theory and research regarding general principles of human behavior in organizational environments [38].

### Effect of Student SES in Medical School

While other social theories focus on the individual or environment, PE fit theory is unique in its emphasis on the relationships between the two [39]. Researchers note that these relationships are complex and can change over time, due to changes in individuals and/or changes in their environments [39].

The dynamics of fit are affected by the power imbalance between individuals and institutions. The bulk of evidence from career outcomes research is that the fit dimensions on the organizational side are substantially more important than those on the individual side [5, 40]. Burnout, stress, physical health, cognitive function, and even “flow” in problem-solving are all affected by powerful organization-level patterns [41–44]. These patterns tend to systematically advantage those in traditionally overrepresented groups by poorly fitting those who have been historically underrepresented [45, 46]. Based on PE fit theory and prior studies, lower-SES students should be expected to experience educational difficulties related to disproportionate lack of networks, information, and financial resources. The present study applies and extends PE fit theory in the context of US medical education.

## Method

This study examined the effect of SES on medical education in the context of person-environment fit theory by interviewing students from lower-SES backgrounds on their experience in medical school.

This study was approved by the Institutional Review Board (IRB) at Yale’s School of Medicine in New Haven, CT (Yale myIRB#2000028071). Rooted in constructivist grounded theory, we conducted a qualitative study using semi structured (open-ended) interview questions [47]. This theory-building research approach guided our data collection, analysis, coding, and interpretation of codes/themes. The research team comprised four medical school trainees and six faculty representing seven medical schools. The team was comprised of researchers with professional and academic expertise in medical education. All members have responsibility for supporting first-generation and lower-income medical students or personally identify as members of the study population. This qualitative study followed the Standards for Reporting Qualitative Research (SRQR) reporting guidelines [48].

### Study Population

Study recruitment and enrollment occurred between November 2021 and March 2022. We recruited participants through social media and emailed announcements sent to listservs targeted to first-generation college graduates. Each announcement contained a link to an online study demographic screener. Purposive sampling [49] was used to achieve the broadest diversity of participants possible for our study. Upon clicking the link to the study screener, the prospective participants were invited to complete a Qualtrics [50] demographic survey after providing informed consent to participate. The demographic data for each prospective participant were used to determine study eligibility and collect the participants’ self-reported demographic information (e.g., race/ethnicity).

### Interview Guide

We constructed a semi structured interview guide based on past qualitative studies, focusing on the impact of socioeconomic status on perceptions of medical school experiences (RR, TW, HM). We specifically included a series of open-ended questions. One researcher piloted the interview script using critical theory [51]. The interview guide can be found in Appendix A.

### Procedures

Two research team members (HM and TW) conducted most of the open-ended interviews. Each interview was conducted via Zoom web conferencing technology [52] and lasted 50–60 min; each interview was audio-recorded, transcribed verbatim, and de-identified to fully protect anonymity and confidentiality. The transcribed data were then coded to categorize common themes. When the research coders decided that no new themes could be identified and thematic saturation [53] had been reached, coding of the participant data was ended. Each study participant received a $20 electronic gift card in appreciation.

### Codes and Themes

We used the constant comparative method to analyze and interpret the data [53]. Codes and themes were developed inductively from the data collected. Five team members (HM, TW, RR, MN, DB) met biweekly to review their coding choices on two to three transcripts, share reflections on thematic determinations, and iteratively revise the codebook until all of coders agreed on the final iteration. Each transcript was coded by at least 1 trainee (CH or AP) and 1 medical school faculty member (TW or HM). Coding disagreements were resolved through debriefing until consensus was reached [54]. One researcher (TW) did not participate in this PE fit analysis and writing.

Codes for this analysis were grouped into themes related to the theory of PE fit, including good or poor fit, feeling like an outsider or different, and experiences of cognitive conflict, imbalance, or disequilibrium. We then analyzed relationships between themes by iteratively focus-coding, re-sorting, and refining groupings of representative statements within the identified PE fit excerpts. Subthemes were identified and conceptually organized to illustrate relationships.

As described in a book on grounded theory by Glaser and Strauss [49], trustworthiness of the thematic findings was enhanced through coder self-reflection during data collection and analysis, in tandem with our own diverse professional and personal backgrounds/perspectives [49]. Reflective practice among the coding, analysis, and writing teams was formally embedded in meetings. Member checking was conducted with a subset of study participants to enhance confidence in interpretations and summary findings. We used Dedoose version 9.0.17 to analyze the qualitative data [55].

## Results

Of 116 prospective first-generation college and/or low-income student participants who responded to the recruitment announcement, we were able to contact 67 via email and subsequently interviewed 48. These medical students represented 27 medical schools across 16 states. Fourteen were at private and the other 13 were at public medical schools. Six students identified as first-generation college graduates only; 12 identified as from a lower-income family background only; and 30 identified as both first-generation and lower-income. Of the 48 participants, 28 identified as female. Twelve identified as White; 12 as Black or African American; 12 as Hispanic, Latino, or of Spanish origin (reported here as primary category rather than overlapping); 11 as Asian or Asian American; 1 as American Indian/Alaska Native; and 1 as Middle Eastern/North African. Table 1 shows the demographic breakdown of interview respondents included in this study.

**Table 1:** Participant demographic characteristics among medical students who completed interviews about professional identity formation in November 2021–April 2022 (*N* = 48)

Characteristics	Number	Percent
		(*N* = 48)
**Sex**		
Female	29	60.4%
Male	19	39.6%
**Race/ethnicity**		
American Indian or Alaskan Native	1	2.1%
Asian or Asian American	10	20.8%
Black or African American	12	25.0%
Hispanic, Latino, or of Spanish Origin	12	25.0%
Middle Eastern North African	1	2.1%
White, Non-Hispanic	12	25.0%
**Combined underrepresented in medicine**		
URiM: Black, Hispanic, or American Indian	25	52.1%
**Low-income and first-generation backgrounds**		
Low-income background only	11	22.9%
First-generation college student only	6	12.5%
First-generation college student and low-income background	31	64.6%
**Highest level of education completed by primary parents/parental figures**		
Elementary	4	8.3%
Some high school	1	2.1%
High school graduate/GED	23	47.9%
Associate degree or equivalent	4	8.3%
Some college, no Bachelor’s degree	4	8.3%
Bachelor’s degree	10	20.8%
N/A	2	4.2%
**Program type**		
MD	46	95.8%
DO	2	4.2%
**School type**		
Public	13	48%
Private	14	52%
**Phase of school**		
Clinical	29	60.4%
Pre-clinical	19	39.6%
**Age**		
18–24	1	25.1%
25–29	7	54.1%
30 and older	10	20.8%
**Family income growing up**		
$0 – $24,999	12	25.0%
$25,000 – $49,999	22	45.8%
$50,000 – $79,999	7	14.6%
$80,000 – $119,999	4	8.3%
$120,000 – $199,999	1	2.1%
Do not know	1	2.1%
Prefer not to answer	1	2.1%
**SNAP or federal assistance programs growing up**		
Yes	23	47.9%
No	20	41.7%
Do not know	3	6.3%
Prefer not to answer	2	4.2%

Interview subjects described experiences related to PE fit in 3 broad domains: (1) school environments, (2) clinical environments, and (3) professional environments. Each of these aspects of medical education includes its own challenges and expectations that may be aligned or misaligned for individual learners. Medical students from lower-SES backgrounds describe struggles to navigate these multiple environments that are unfamiliar, culturally complex, and both personally and financially costly. They also describe ways they are addressing gaps in healthcare and education, generating positive changes at their schools, supporting underserved patient populations, and broadening perspectives of peers and educators.

### School Environments

Important concepts related to medical school environments were (1) need for additional organizational support, (2) concerns regarding costs, (3) performance assessments, (4) curricular content related to underserved populations, (5) representation, (6) negative responses from others, and (7) difficulties experienced. Many of the factors are amenable to organizational intervention. Students reported variable recognition and support for lower-SES learners across their medical schools. Selected quotes representing these issues are provided below:

- Organizational supports
  We want to increase the diversity in our medical school and in our residency, but with increasing diversity comes the adversity of being diverse…. It’s not just a pretty thing to put in a report. So, we increased on Latinos by this many. We increased our African American students by this many. We require resources, very specific helps, from very specific people that understand how to help us. (ID_38)
- Cost
  Money is the #1 thing…. That’s the biggest stressor. When you are wondering whether or not you’re going to be able to make it to the end of the year paying rent…. My parents had been diagnosed with COVID, my dad was not well, they hadn’t been working for months…. There’s a huge disconnect between administrators and their understanding of what poor students go through. (ID_20)
- Assessment
  Step 1 is now pass-fail…. [That] can be good and bad. Good in the sense that there’s a lot less pressure to kind of have that numerical score. But more negatively, I’m thinking about residency applications. In the past, it’s always been, your Step 1 score and your Step 2 score really make a difference in which specialties [you can pursue]. And I think now it’s more about the networking. And I think that is where being first-gen, low-income can really be taking a hit. Because it’s already a struggle trying to find mentors and physicians like myself that can help me mentor along the way. (ID_23)
- Curriculum/teaching
  I felt very out of place in that setting because it’s something that I had never experienced in my life before. But then also because we spent the day talking about how many members of the community around the institution are suffering from low financial means or from not enough social services, and then we flip the page and that night we kind of indulge in these kinds of extravagancies. (ID_26)
- Representation
  Being a Black male, especially trying to be a physician, it has its pros and cons…. You kind of have to be the face for the people coming behind you. That’s a lot how it may feel at times… because there’s so little of us in the first place. And so anything you do is kind of magnified. (ID_37)
- Response from others
  I don’t see my life as something to be ashamed of. The only time I questioned what to reveal was when I got to medical school. I wasn’t sure if I should tell people that I had been in a program that helped me get there…. There are some people that feel like programs let people into med school that don’t belong there or who’ve got in the quote-unquote ‘easy way.’ So people either say that or people will say, ‘Oh, but you were really smart. You would have gotten into medical school anyway. You’re the exception to that stereotype.’ And I didn’t really want either of those opinions. (ID_18)
- Difficulties
  There’s this low-dose anxiety that keeps following me as I go through all this. And it just tells me like, ‘Hey, [redacted], you can’t be like other people. Other people got the money, the time, the resources. You can’t. You have to secure this for yourself. Nobody else is going to do this for you’…. That kind of restricts me or pulls me back from getting more and new experiences. (ID_43)

### Clinical Environments

Reflections on clinical environments included repeated comments about fitting or not fitting regarding (1) patient care settings, (2) specialty expectations, (3) patient populations, and (4) clinical cultures. Responses in this domain highlighted that lower-SES medical students feel they fit within a role of serving patients who reflect their families.

- Patient care
  Clinics are my thing. That’s why I’m going into family medicine, specifically to go into Federally Qualified Health Centers and underserved kind of clinics. That’s where I belong. I don’t belong in a hospital. Don’t put me in the ICU. So yeah, it felt to me like I belong there. (ID_38)
- Patient populations
  I guess I see medicine from… more the patients’ view…. Right now, I’m working in the county system…I feel like I have more in common with them… than I do the doctors. (ID_9)
- Specialties
  [My background] really affected which career seemed like an option for me. The surgical subspecialties and the specialties that can make the most money just didn’t seem accessible…. I’d consider them for a second and then felt like, ‘Wow, I’m dumb for considering this. I don’t belong here. Never mind. Forget it. I don’t fit there.’ (ID_30)
- Clinical cultures
  You hope to never lose sight of where you’re from, how people will see you and see your culture differently than their own. (ID_42)

### Professional Environments

Reflections on professional environments include comments about (1) role models, (2) feeling like an outsider with peers, (3) feeling a sense of belonging with peers, and (4) fitting or misfitting with definitions of being under-represented in medicine (URiM). Medical students navigate a broader community environment throughout their educational careers. Their relationships with others in the professional community, as well as their personal fit with community values, powerfully shapes their sense of belonging in medicine and specific subfields.

- Role models
  I remember working in the free clinic my first year of medical school, there was a resident who said “Oh, my dad used to be a patient at this free clinic.” Just hearing those words, seeing someone who’s a doctor and whose dad was accessing healthcare in a similar way to my parents at one point in their lives, meant a lot. And it made patients feel less othered. (ID_1)
- Peers: Outsider
  You see students who are able to take summers off. They don’t have to work or go into research for the stipends. Or people who have doctors as parents, I think they kind of have that thing that is passed down to them. They know kind of what to expect. They have more information. [ … ] I have this one friend, from a fairly wealthy family—her family threw a party for us at a restaurant. I couldn’t even imagine how much it cost, but it was for like 40 people. (ID_31)
- Peers: Belonging
  I would tell [new medical students] to make sure they find a community, make sure they find people who have their back, people who understand where they’re coming from, whether that be people from the same background or allies, and just make sure they also stay connected to their roots in terms of home, if that’s something that’s really important to them. I feel like you kind of lose yourself sometimes in medicine because you’re changing from the community grew up in but you don’t necessarily fit in into medicine either, so it’s kind of a weird balance…overall I would just tell them that they’re needed in medicine, and that I’m glad that they’re there. (ID_41)
- Peers: URiM definitions
  I get lumped into a pool of people that are very well-off, that are very privileged. I acknowledge those things, the ones that I do have. But it does make it harder when you’re lumped into a group like that, and you’re not like that. (ID_22)

**Fig. 1:**
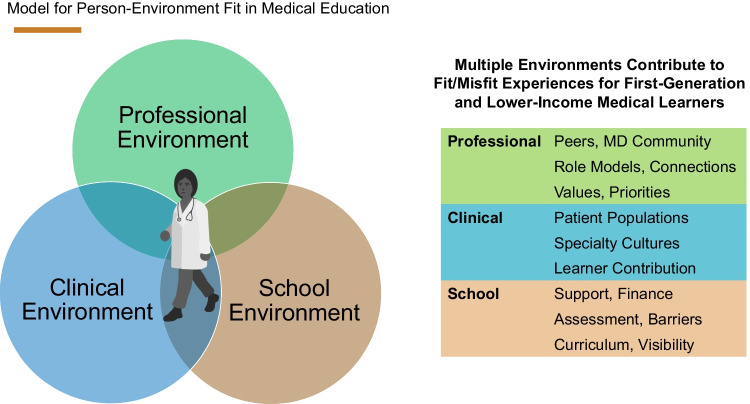
Theoretical model of interrelated environments influencing PE fit for lower-SES medical students (doctor graphic: E. Riley)

Though helpful for analytical purposes, these categories overlap in the experience of medical students. They must learn to navigate school, clinical, and professional environments simultaneously to become effective physicians. Figure 1 depicts these interacting environments and their relevant components.

Though this study focused on SES, it was evident that some students’ experience of fit or misfit was based on other characteristics or aspects of their identity. For example, race/ethnicity, gender, sexual orientation, and disability were also critical to understanding the experience of many of these students [56]. Medical students with multiple socially and economically marginalized identities must navigate multiple axes of power dynamics. The ways in which they experience fit or misfit with their environments will depend on the characteristics of their school, clinical, and professional environments. Specifically, the success of first-generation and low-income medical students is shaped by the ability of medical schools, professional peers, and the community of medicine to understand and fully incorporate their diversity.

### Limitations

The most important limitation is regarding representativeness in the study population. Firstly, the medical students who chose to complete interviews may differ in important ways from the full population of students from first-generation college and low-income backgrounds. These students may not fully represent the range or intensity of experiences. Secondarily, some key study personnel did not attend medical school, which could limit their interpretations.

## Conclusions

Medical students from lower-SES backgrounds have different pressures and priorities in professional identity formation than their peers and will experience profound SES changes relative to their families of origin. It is critical for medical schools, funders, peers, and professional communities to sustain learning environments that support these students’ flourishing. This includes transferring the burden of addressing “fit” from individual learners to educational, clinical, and professional organizations.

### Professional Gaps

Medical schools and professional groups have a special obligation to be responsive to student populations who have been recruited to address a specific need. One important role for medical education leaders is to develop strategies to maximize the full potential of first-generation and lower-income medical student population, while mitigating known structural challenges [56, 57]. When we focus on cultivating these medical students, trainees, faculty, and staff, we are taking steps to improve the future state of healthcare delivery for all our patients.

Institutional change must involve efforts to attract diverse students to all subspecialties and lessen barriers to entry. Additionally, schools and the wider profession of medicine should recognize and reward paths that include working with underserved populations and communities, a clinical context in which many of these learners report feeling most comfortable, seen, and purposeful.

Students’ interest in serving the underserved could be supported by creating medical school environments that explicitly offer and affirm community-engaged and community-centered medicine. Revising curriculums to be more interdisciplinary, especially integrating insights from social science and public health, could result in more complex discussion, analysis, and understanding of social and economic aspects of health among students.

### Support Needs

Programming to recruit more diverse populations without adequate support systems continues to put a strain on these students. Greater involvement of lower-SES voices as stakeholders in programming efforts will contribute to better shared solutions. Institutions must recognize the diversity within the first-generation and lower-income student populations. Lower-SES medical students are disproportionately from racial and ethnic groups that have a lower proportion of doctors relative to patient populations.

Students expressed a need for empathy and attention to their practical concerns, rather than sympathy or pity. Many of these students continue to perceive a lack of accessibility to the same resources available to students from more advantaged backgrounds. Providing quality mentorship for these students is also key.

### Professional Identity Negotiation

The medical school journey involves ongoing efforts to negotiate professional identity and navigate unfamiliar environments. While some students can feel socially, academically, and professionally at odds with the norms of medical culture, they also adapt their sociocultural identities to those norms. Students’ negotiation of identity reinforces that PE fit is dynamic. Individual students may need different kinds of support at different times during their education and training.

To best support learner development, future medical school policies and programs should recognize that students undergo a negotiation and adaptation process during the formation of a coherent professional identity. This process of finding equilibrium within new environments can be more or less challenging for different learners at different times. To encourage individual agency in the developmental process, schools and clinical learning environments should offer structures that encourage students to capitalize on these experiences of cognitive disequilibrium for personal growth (with appropriate just-in-time support).

### Managing Fit

The interview results indicate that schools can do more to explore “fit” with these students when they start their medical journey. It could help for them to verbalize and unpack their experiences so that they can process these feelings before they become internalized into negative recurrent thoughts (e.g., imposter syndrome). We wonder if this type of cognitive processing should be part of routine curriculum for first-generation and low-income learners and trainees, especially when studies like this highlight how impactful environment and context are to the education journey.

Personal background is an important facet of the conversation as students consider specialty career choices. Schools, clinical training sites, and professional societies should amplify the toolkit for these students to manage environments and specialty cultures that are far from their home “fit.” Trained mentors can help learners reflect on why they are pursing or avoiding a given specialty.

Fit management strategies should focus on increasing opportunities to develop cohesive groups as well as cohesion across groups. Additionally, students repeatedly asked for more supportive and reflective role models. They may benefit from mentorship opportunities with members of the community, peers, or faculty/staff with similar backgrounds and experiences. Cognitive conflicts and emotional disequilibrium are difficult experiences. Institutions can focus on providing emotional as well as academic or financial support in medical school, including educational counseling services.

This research and discussion lead us to consider more seriously the professional gaps that exist in medical education and the practice of medicine. Those persistent gaps affect groups differently and highlight the urgent need to address educational supports, the negotiation of professional identities, and ongoing management of fit as shared foci for learners and institutions. Medical students from underrepresented backgrounds face many cognitive conflicts and intense periods of disequilibrium as they navigate unfamiliar environments in medical school and residency. Medical schools, clinical environments, and professional societies can cultivate environments where diverse learners are well-supported and feel they belong. Accrediting bodies could help shine a light on these issues by focusing attention on feedback from learners, faculty, and staff on their experiences of fit and expanding programs that encourage belonging for the widest range of people.

## Supplementary Information

Below is the link to the electronic supplementary material.

Supplementary file1 (DOCX 24.4 kb)

## Data Availability

The datasets generated and/or analyzed during the current study are not publicly available due to confidentiality protections. The approved IRB protocol requires that the transcripts and associated data files be destroyed at the close of the research study to protect confidentiality of the interview subjects.
