# Supplementary material for: Person-Environment Fit and Socioeconomic Status in Medical School
Source: Med Sci Educ. 2024 Oct 3;35(1):233–43. doi: 10.1007/s40670-024-02174-x (PMC11933605; doi:10.1007/s40670-024-02174-x)
Supplement: Supplementary file 1 — Supplementary file1 (DOCX 24.4 kb) [file 40670_2024_2174_MOESM1_ESM.docx]

**Supplemental Digital Appendix 1**

Interview questions from a national, US-based, study of the experiences of first-generation college graduate and/or low-income medical learners. 2021-2022.

1. Tell me a little bit about experiences that shaped your pursuit of a career in medicine.
2. Do you consider yourself as a First-Generation college graduate, from a Low-Income background or Both? In what ways has your FG and/or LI background been an asset during medical training?
3. What sacrifices, as an FG/LI student, have you made in medical school?

1. **M4s only**: Can you share a bit about how the application to residency and MATCH process has been for you?
2. What kinds of things take up your time as a medical student that perhaps is not the case for peers who aren’t from low-income and/or first-generation college graduate backgrounds?
3. How would you define the culture of medicine?
4. Do you have a story about a time in medical school when you felt like an *outsider, misunderstood, off balance or knocked back etc,* because of your FG and/or LI background?

1. What aspects of being an FG and/or LI student do you not share with others unless you trust them?
2. How have your perceptions of yourself as a physician in training changed as you’ve gone through medical school? How has that affected you?
3. Do you feel you belong, are visible, safe and supported and that your school cares about your success as an FGLI medical student?
   1. **If not**, what could faculty, staff and/or your school in general, do to help you feel like you belong are visible, safe and supported?
   2. **If so**, what do faculty and/or your school do that make you and students like you feel you belong, are visible, safe and supported as a student from a FG and/or LI background?
4. What have been the most important supports for you as you’ve navigated medical school? What supports that are not at your school now, but you wish were?
5. What concrete steps can medical school administrators and faculty take to promote the development of future physicians from backgrounds similar to yours?
6. Thinking about learners from similar backgrounds like yours - what advice would you give about navigating:
   1. the pre-medical school pathway?
   2. medical school?
7. Do you think your FG and/or LI background will impact your future career path? If so, how so?
8. What will it mean when you can say ‘I am a physician.’?
